# 19q13 KRAB zinc-finger protein ZNF471 activates MAPK10/JNK3 signaling but is frequently silenced by promoter CpG methylation in esophageal cancer

**DOI:** 10.7150/thno.35861

**Published:** 2020-01-12

**Authors:** Ran Sun, Tingxiu Xiang, Jun Tang, Weiyan Peng, Jie Luo, Lili Li, Zhu Qiu, Yiqing Tan, Lin Ye, Min Zhang, Guosheng Ren, Qian Tao

**Affiliations:** 1 Key Laboratory of Molecular Oncology and Epigenetics, The First Affiliated Hospital of Chongqing Medical University, Chongqing, China; 2 Cancer Epigenetics Laboratory, Department of Clinical Oncology, State Key Laboratory of Translational Oncology, Sir YK Pao Center for Cancer and Li Ka Shing Institute of Health Sciences, The Chinese University of Hong Kong and CUHK-Shenzhen Research Institute, Hong Kong; 3 Department of cardiothoracic surgery, The First Affiliated Hospital of Chongqing Medical University, Chongqing, China

**Keywords:** ZNF471, CpG methylation, MAPK10/JNK3, zinc finger protein, esophageal

## Abstract

Zinc-finger proteins (ZFPs) are the largest transcription factor family in mammals, involved in the regulation of multiple physiologic processes including cell differentiation, proliferation, apoptosis and neoplastic transformation. Approximately one-third of ZFPs are Krüppel-associated box domain (KRAB)-ZFPs.

**Methods**: ZNF471 expression and methylation were detected by reverse-transcription PCR and methylation-specific PCR. The impact and mechanism of ectopic ZNF471 expression in esophageal squamous cell carcinoma (ESCC) cells was evaluated in vitro and in vivo.

**Results**: We identified a 19q13 KRAB-ZFP, *ZNF471*, as a methylated target in ESCC. We further found that ZNF471 is significantly downregulated in ESCC tissues compared with adjacent non-cancer tissues, due to its aberrant promoter CpG methylation, and further confirmed by methylation analysis and treatment with demethylation agent. Restoration of ZNF471 expression in silenced ESCC cells significantly altered cell morphology, induced apoptosis and G0/G1 arrest, and inhibited tumor cell colony formation, viability, migration and invasion. Importantly, ZNF471 was found to activate the expression of *MAPK10/JNK3* and *PCDH* family genes, and further enhance MAPK10 signaling and downstream gene expression through binding to the *MAPK10/JNK3* promoter.

**Conclusion**: Our results demonstrate that ZNF471 is an important tumor suppressor and loss of ZNF471 functions hampers *MAPK10/JNK3* signaling during esophageal carcinogenesis.

## Introduction

Esophageal cancer is an aggressive cancer, and is the eighth most common cancer and the sixth leading cause of cancer-related mortality worldwide 1. Approximately 70% of global esophageal cancer cases occur in China 2, and 90% of cases are esophageal squamous cell carcinoma (ESCC) 3. China has the highest ESCC risk worldwide. The incidence of ESCC has recently slightly decreased, but its 5-year survival still remains poor. Thus, better understanding of its pathogenesis is urgently needed 4-8. Although the molecular mechanisms underlying esophageal carcinogenesis are largely unknown, epigenetic silencing of key tumor suppressor genes (TSGs) by promoter CpG methylation has been demonstrated to be critical for esophageal tumorigenesis 9, 10. Therefore, identification of novel TSGs inactivated by DNA methylation would be useful to establish biomarkers for ESCC.

Zinc-finger proteins (ZFPs) are the largest transcription factor family in mammals. Their zinc-finger domains bind to gene promoters and consequently activate or repress the expression of target genes 11. Nearly one-third of ZFPs contain a highly conserved Krüppel-associated box (KRAB) motif, which contributes to transcriptional repression through recruitment of histone deacetylase (HDAC) complexes 12-15. A large number of ZFPs are located in several gene clusters at the long arm of chromosome 19. Deletion of 19q is frequent in multiple cancers including ESCC, cervical cancer, and nasopharyngeal carcinoma 16-18. Previously, our group has identified and defined several KRAB-ZFP proteins, including ZNF382, ZNF545, and ZNF331, as important novel TSGs frequently downregulated and methylated in carcinomas 11, 19-24. We also identified ZNF471, another member of the KRAB C2H2-type ZFP family. Frequent methylation of ZNF471 in cancer has been reported in colorectal and squamous cell carcinoma 25, 26, and its biological functions have been reported to be involved in the regulation of cell proliferation, apoptosis, migration and invasion, as a tumor suppressor in gastric cancer 27. However, its abnormalities, biological functions and underlying mechanism in ESCC remain unknown.

In the present study, we investigated the expression and promoter methylation status of ZNF471 in primary ESCC and its biological functions. We further explored the molecular mechanisms of its tumor suppression in ESCC cells.

## Results

### ZNF471 is downregulated by promoter CpG methylation in ESCC cell lines

**Figure 1:**
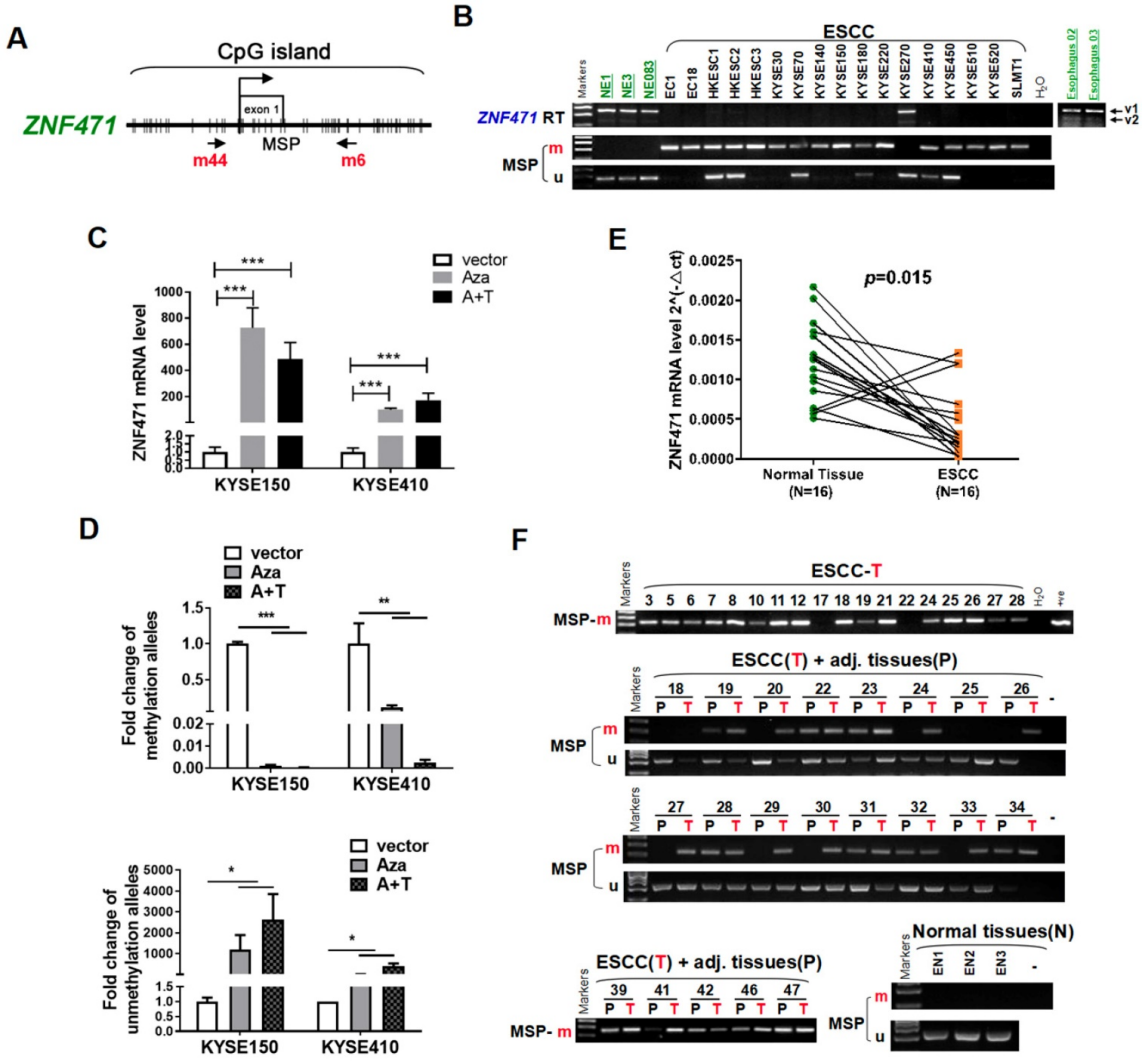
** Identification of ZNF471 silenced by promoter methylation in ESCC cell lines.** (A) A typical CpG island spanning ZNF471 (CpG Island Searcher). Each vertical bar represents a single CpG site. (B) ZNF471 expression and methylation status in ESCC cell lines. The RNA integrity of these samples was confirmed by GAPDH tests, as shown in our other publications 20. M, methylated; U, unmethylated. (C, D) ZNF471 expression and methylation status with 5-aza-2-deoxycytidine (Aza) and trichostatin A (TSA) treatments in ESCC cell lines. Demethylation was measured by real time quantitative MSP (qMSP). M, methylated; U, unmethylated. Dunnett's t-test was used. (E) ZNF471 expression in primary ESCC (n=16) and paired adjacent non-cancerous tissues (n=16) by qRT-PCR. Student's test was used. Data are presented as the mean ± SD. (F) ZNF471 methylation in primary ESCC tissues (n=147), adjacent non-cancerous tissues (n=89) and normal tissues (n=3), measured by MSP. M methylated, U unmethylated. Gel images showed were just representational graphs, not for all gel images. *p<0.05, **p<0.01, ***p<0.001.

We first examined the expression and promoter methylation of *ZNF471* in normal esophageal tissues and ESCC cell lines. ZNF471 has two isoforms (isoform 1: NM 020813.4; isoform 2: NM 001321768.2), with its short isoform 2 lacking the KRAB motif. Sequence analysis by CpG Island Searcher revealed that the ZNF471 promoter contains a CpG island (Fig. 1A), thus indicating that CpG methylation may be a major mechanism regulating its expression 28. By semi-quantitative RT-PCR, we found that ZNF471 expression was silenced in most ESCC cell lines but highly expressed in immortalized epithelial cell lines (NE1, NE3 and NE083) and normal esophageal tissues (Fig. 1B). Even in normal tissues and cell lines, the short isoform 2 was barely detectable; thus, we mainly further studied the functions of isoform 1, referred to as ZNF471 herein. Further methylation-specific PCR (MSP) analysis showed that the ZNF471 promoter was methylated in 16/17 (94%) ESCC cell lines (Fig. 1B), a finding correlated with its downregulation. In contrast, no methylation was detected in immortalized normal epithelial cell lines (Fig. 1B).

To determine whether CpG methylation directly mediates ZNF471 downregulation, we treated the ESCC cell lines KYSE150 and KYSE410 with the DNA methyltransferase inhibitor Aza, alone or together with the HDAC inhibitor TSA. This treatment restored ZNF471 expression, accompanied by a decrease in methylated promoter alleles and an increase in unmethylated alleles (Fig. 1C, D). Together, these results indicate that promoter CpG methylation of ZNF471 is a mechanism contributing to its downregulation or silencing in esophageal cancer.

### ZNF471 is downregulated by CpG methylation in ESCC tissues

We examined ZNF471 expression by real-time RT-PCR in paired esophageal tumor and adjacent non-tumor tissues. ZNF471 expression was downregulated in 87.5% (14/16) of the primary tumors, compared with corresponding adjacent tissues (*p*=0.015, Fig.1E). Thus, we identified ZNF471 as a frequently downregulated gene in ESCC.

To examine its promoter methylation status, we performed MSP analysis on 147 primary ESCC tumors, including 89 paired and 58 tumors, and 3 normal esophageal tissues. ZNF471 methylation was detected in 92/147 (62.6%) tumors but only 22/89 (24.7%) paired adjacent non-tumor tissue samples, and 0/3 (0%) normal esophageal tissues (Fig. 1F) (Table 1), thus suggesting that ZNF471 methylation is a common event in ESCC. Next, through The Cancer Genome Atlas (TCGA) Research Network, the relationships between ZNF471 methylation and patient clinicopathological features were analyzed, including sex, age, tumor stage, tumor size, lymph node metastasis, and tumor differentiation. However, no significant correlation between ZNF471 methylation and clinicopathological features was found. (Supplementary Table 1). In addition, we used the same tissue samples from TCGA Research Network to analyze the correlation between ZNF471 expression and methylation and found that they were negatively correlated (r=-0.4128, *p*<0.0001) (sFig. 1).

### ZNF471 suppresses ESCC cells growth

**Figure 2:**
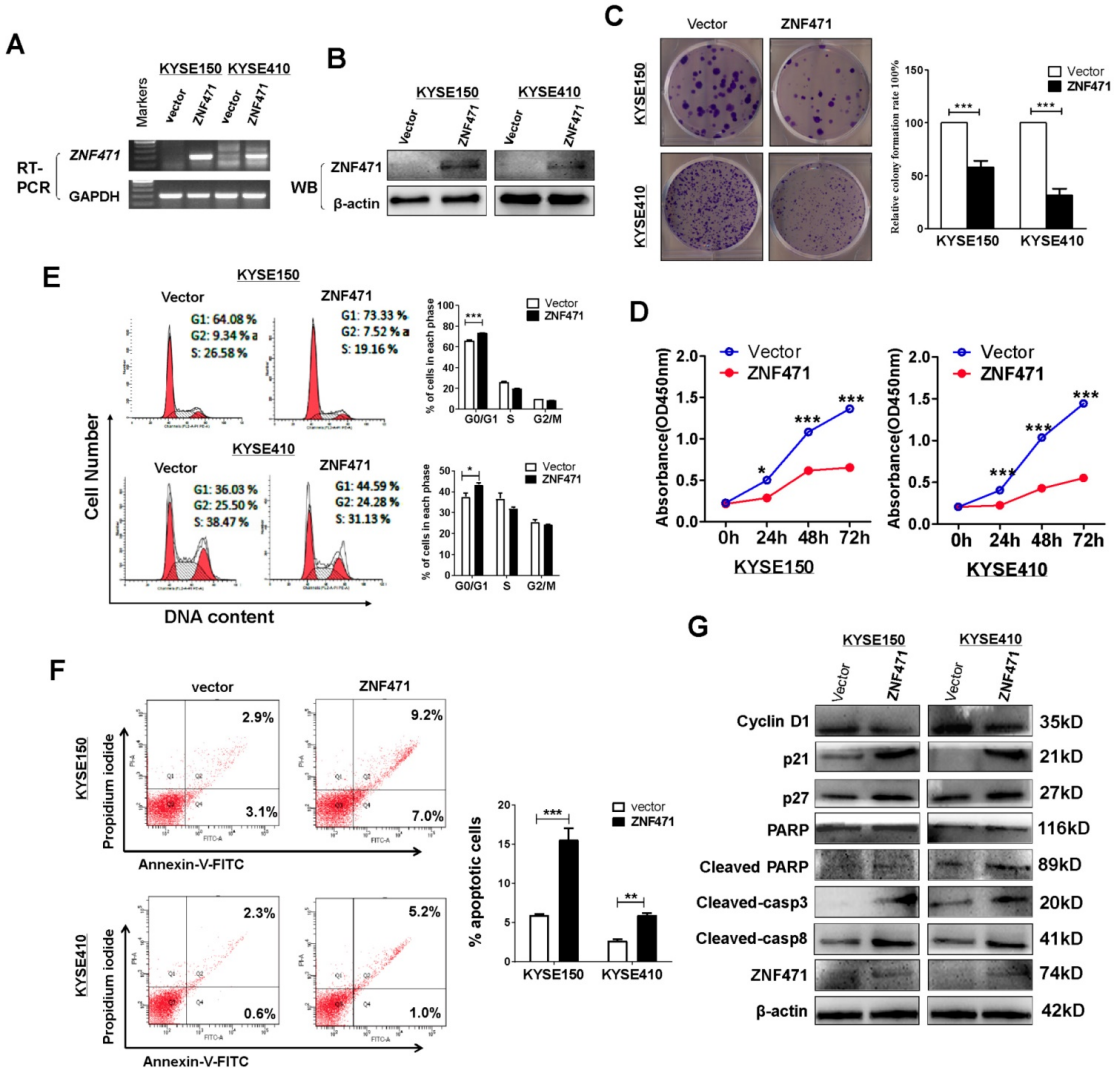
** ZNF471 suppresses ESCC cell proliferation and induces G0/G1 arrest and apoptosis.** (A, B) Ectopic expression of ZNF471 in ESCC cell lines was measured by RT-PCR and western blotting. (C) Colony formation of vector- and of ZNF471-transfected KYSE150 and KYSE410 cells (left panel). Rates are shown as mean ± SD (right panel) from three independent experiments. (D) Cell viabilities evaluated at 24, 48 and 72 h after transfection with ZNF471 in KYSE150 and KYSE410 cells. (E) Cell cycle distribution measured in vector- and ZNF471-stably transfected KYSE150 and KYSE410 cells by flow cytometry. Representative distribution plots and histograms of alterations are shown. (F) Percentages of apoptotic cells in KYSE150 and KYSE410 cells with ZNF471 ectopic expression were evaluated. Cell apoptosis alterations were revealed by histograms. (G) Expression of Cyclin D1, p21, p27, cleaved -caspase 8, -caspase 3 and -PARP measured by western blotting in ZNF471-transfected ESCC cells. Student's test was used. Data are presented as the mean ± SD. *p<0.05, ***p<0.001.

To assess its tumor suppressive functions, we transfected pcDNA3.1- ZNF471-Flag-V5-expressing plasmid into ESCC cell lines KYSE150 and KYSE410 with silenced expression of ZNF471. Restoration of ZNF471 after stable transfection was verified with RT-PCR (Fig. 2A) and western blotting (Fig. 2B). ZNF471 expression was found to suppress the growth of both KYSE150 and KYSE410 cells (*p*<0.001) in colony formation assays (Fig. 2C) and cell viability assays (Fig. 2D). The viability of ZNF471-expressing ESCC cells was significantly lower (*p*<0.001) than that of vector control cells (Fig. 2D).

### ZNF471 induces cell cycle arrest and apoptosis in ESCC cells

To explore the underlying mechanism through which ZNF471 inhibits ESCC cell growth, we analyzed the effects of ZNF471 on cell cycle distribution by flow cytometry using stably expressing cells. Re-expression of ZNF471 significantly increased the proportion of G0/G1 phase cells of the KYSE150 (*p*<0.001) and KYSE410 cell lines (*p*<0.05) (Fig. 2E). Flow cytometry analysis also showed that ZNF471 ectopic expression significantly increased the numbers of apoptotic cells in the KYSE150 (*p*<0.001) and KYSE 410 cell lines (*p*<0.01), compared with vector control cells (Fig. 2F). Western blots showed that ZNF471 significantly increased levels of p21, p27, cleaved caspase 8, caspase 3 and PARP, and decreased expression of Cyclin D1 in ESCC cells compared with vector control cells (Fig. 2G).

### ZNF471 represses ESCC cell migration and invasion through reversing EMT

**Figure 3:**
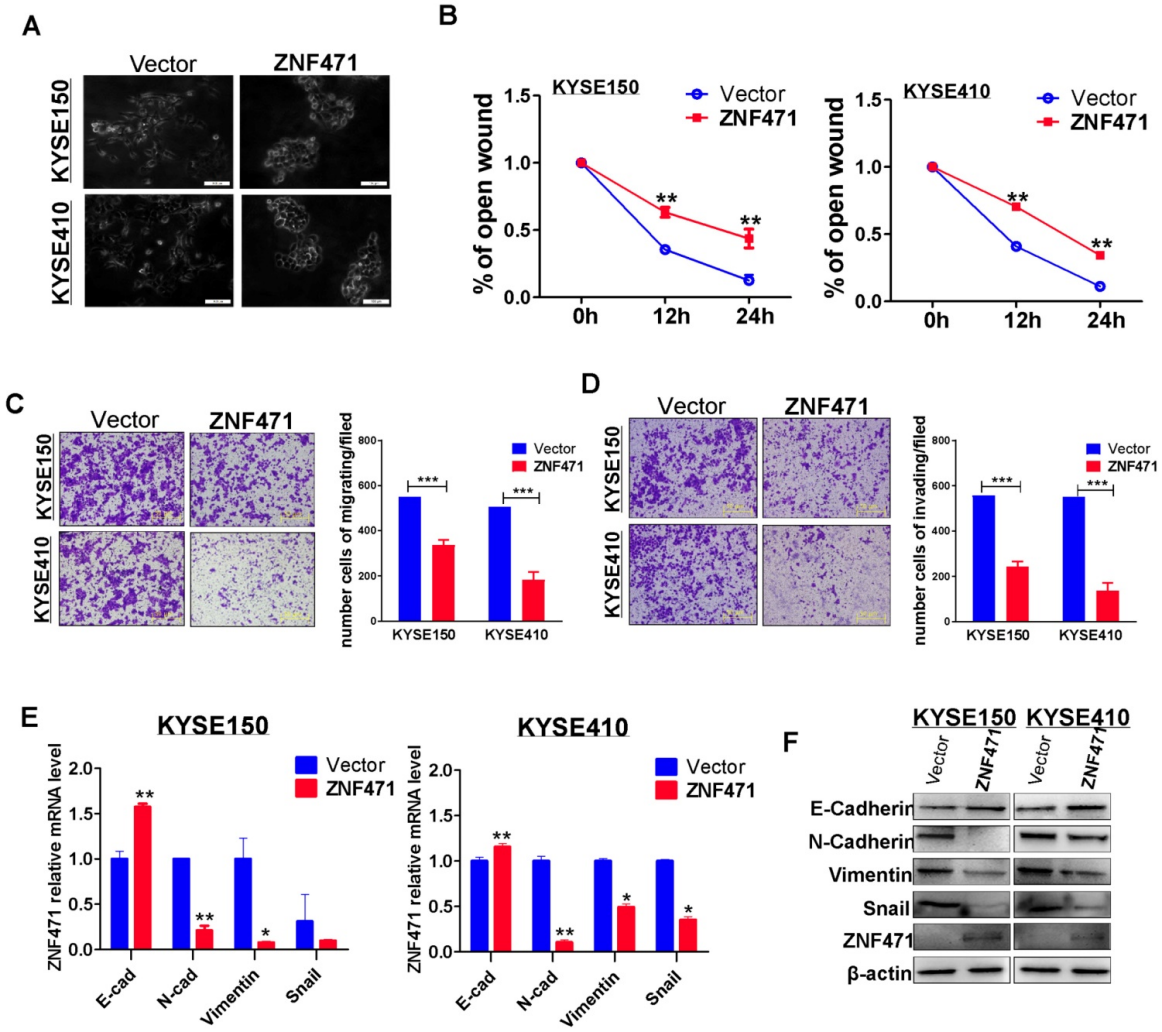
** ZNF471 inhibits ESCC cell migration and invasion and EMT.** (A) Morphological changes of ESCC cells infected with control vector and ZNF471 by phase contrast microscopy. Scale bars: 200 μm. (B) Cell migration abilities of ESCC cells evaluated by wound healing assays. Photographs captured at 0, 12 and 24 h. The cellular migration (C) and invasion (D) abilities of ESCC cells upon ectopic expression of ZNF471 were measured by Transwell assays with or without Matrigel. Representative images were photographed following fixation and staining. Scale bars: 50 μm. (E, F) qRT-PCR and western blot analysis of EMT, and downstream target markers. Student's test was used. Data are presented as the mean ± SD. *p<0.05, **p<0.01, ***p<0.001.

To investigate the effect of ZNF471 expression on ESCC cell migration, we examined cell morphological changes (Fig. 3A). ZNF471 stably transfected ESCC cells showed adherence and contacts with each other; however, vector control cells exhibited a scattered pattern, thus indicating that ZNF471 is likely to be involved in tumor cell epithelial mesenchymal transition (EMT). Thus, we performed wound healing assays. ZNF471 stably transfected ESCC cells migrated into wounded areas more slowly than control cells at 24 h (*p*<0.01) (sFig 2, Fig. 3B). The effects of ZNF471 on ESCC cell migration and invasion abilities were further analyzed by Transwell® assays. The results showed that ectopic expression of ZNF471 significantly decreased the numbers of migrating (*p*<0.001) (Fig. 3C) and invading cells (*p*<0.001) (Fig. 3D).

Real-time RT-PCR and western blotting showed that the inhibitory effect of ZNF471 on ESCC cell metastasis was mediated by the upregulation of E-cadherin (CDH1) and downregulation of vimentin (VIM), N-cadherin (CDH2) and SNAIL1 (Fig. 3E, F). Immunofluorescence staining further showed greater staining of CDH1 and less staining of VIM in the cytoplasm and membranes of ESCC cells with ZNF471 expression, compared with control cells (sFig. 3). These results demonstrated that ZNF471 inhibited the EMT of ESCC cells.

### Knockdown of ZNF471 promotes cell growth and induces metastasis

**Figure 4:**
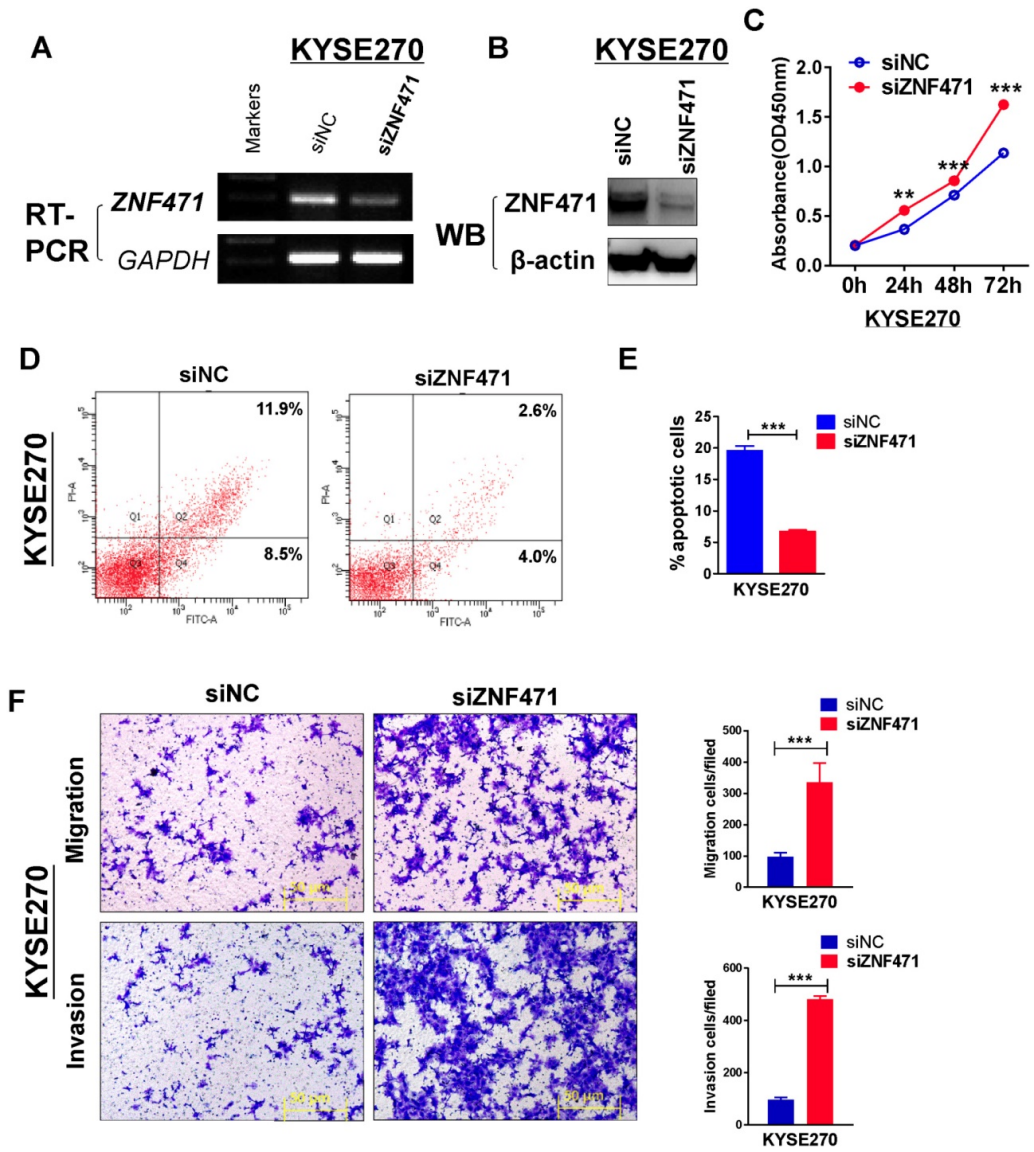
** Knockdown of ZNF471 promotes ESCC cell growth and induces metastasis.** (A, B) Evaluation of ZNF471 knockdown in KYSE270 cells after transfection with ZNF471 siRNA and si-NC by RT-PCR and western blotting. (C) The effect of ZNF471 knockdown on cell viability was measured with cell viability assays. (D, E) Cell cycle distribution after ZNF471 knockdown in KYSE270 cells. (F) Transwell assays showed cell migration and invasion of KYSE270 cells after ZNF471 knockdown. Scale bars: 50 μm. Each experiment was repeated three times. Student's test was used. Data are presented as the mean ± SD.*p<0.05, **p<0.01, ***p<0.001.

To further assess the suppression function of ZNF471 on ESCC cells, we assessed the effect of ZNF471 through siRNA knockdown of ZNF471 in a ZNF471-expressing KYSE270. Knockdown efficiency was detected by RT-PCR and western blotting (Fig. 4A, B). Knockdown of ZNF471, compared with control siRNA treatment, markedly increased KYSE270 cell viability, as examined by CCK8 assays (Fig. 4C). We further used flow cytometry to evaluate the effect of ZNF471 on cell apoptosis. The percentage of apoptosis in KYSE270 cells was significantly decreased after ZNF471 knockdown (*p*<0.001) (Fig. 4D, E). Moreover, we observed enhanced cell migration and invasion after ZNF471 knockdown. The number of migrated and invading KYSE270 cells was dramatically higher after ZNF471 knockdown, compared with that in the control group (*p*<0.001) (Fig. 4F). These knockdown results confirmed that ZNF471 functions as a tumor suppressor in ESCC cells.

### ZNF471 inhibits ESCC xenograft growth in nude mice model

**Figure 5:**
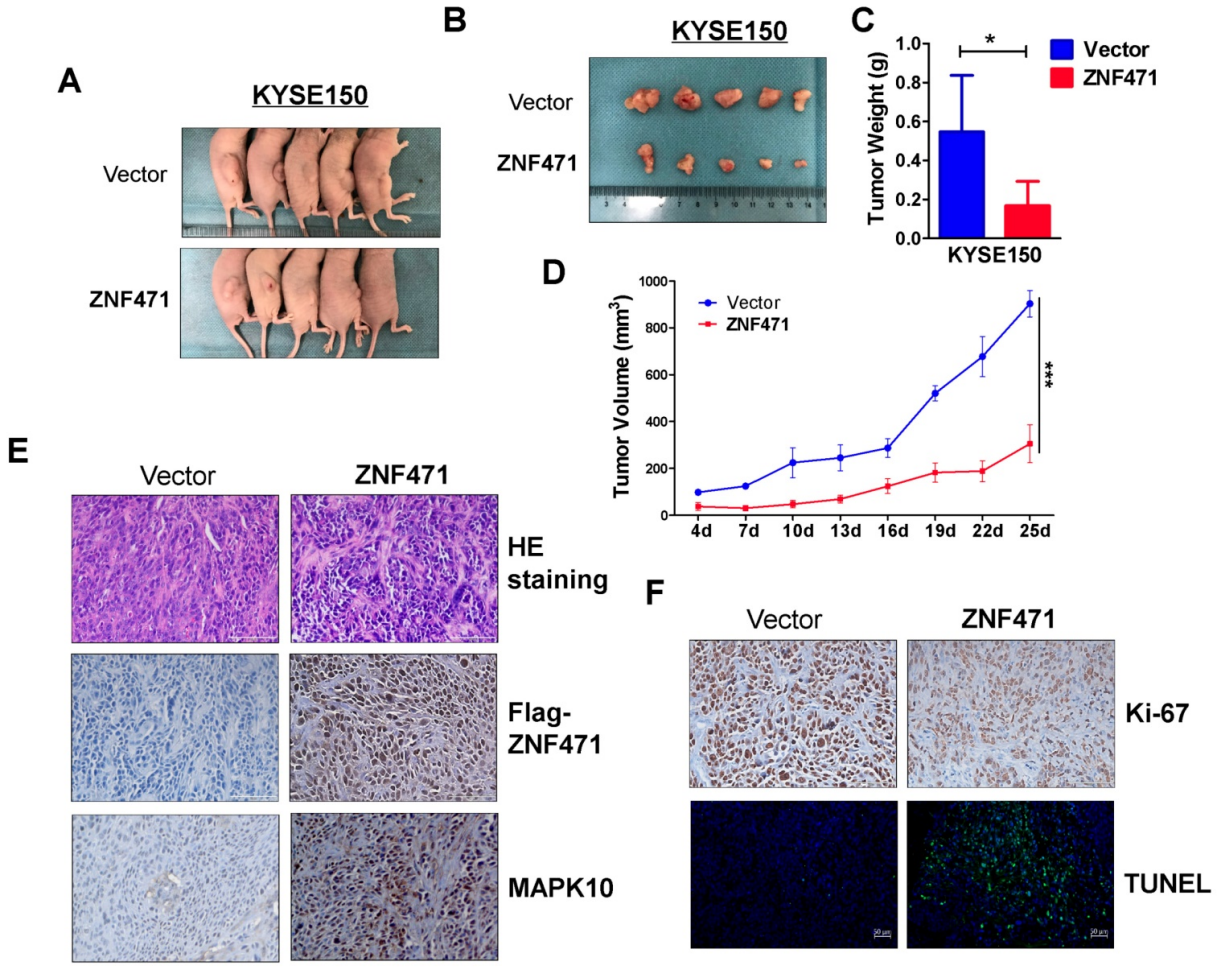
** ZNF471 inhibited ESCC growth *in vivo*.** (A, B) Images of human ESCC tumor xenografts. (C) Comparative histogram of tumor weights in the two groups (empty vector group vs. stably expressing ZNF471 group) of nude mice. (D) Comparative analyses of tumor growth curve for vector- and ZNF471-infected KYSE150 cells in nude mice xenografts. (E, F) Representative photographs of H&E staining and IHC expression analyses of ZNF471, MAPK10, Ki-67, and apoptosis as assessed by TUNEL assays in xenografts. Scale bars: 100 μm. Student's test was used. Data are presented as the mean ± SD. *p<0.05, ***p<0.001.

A nude mouse xenograft model was used to further assess ZNF471 functions *in vivo*. No significant differences in average weight were observed between the two groups of mice (data not shown). The average volume and weight of tumors were significantly lower in the ZNF471 group than the control group (Fig. 5A-D). Immunohistochemistry (IHC) and hematoxylin and eosin staining were carried out to analyze ZNF471 expression and tumor features of the xenografts in nude mice. Ki-67 staining and TUNEL analyses were carried out to evaluate cell proliferation and apoptosis, respectively. Tumor cells with frequent nuclear fragmentation were primarily observed in xenografts with ZNF471 expression, along with increases in MAPK10 staining, decreases in Ki-67 staining and increases in cell apoptosis (Fig. 5E, F). Together, these results indicated that ZNF471 inhibits ESCC tumorigenesis.

### Identification of ZNF471-regulated genes in ESCC cells

**Figure 6:**
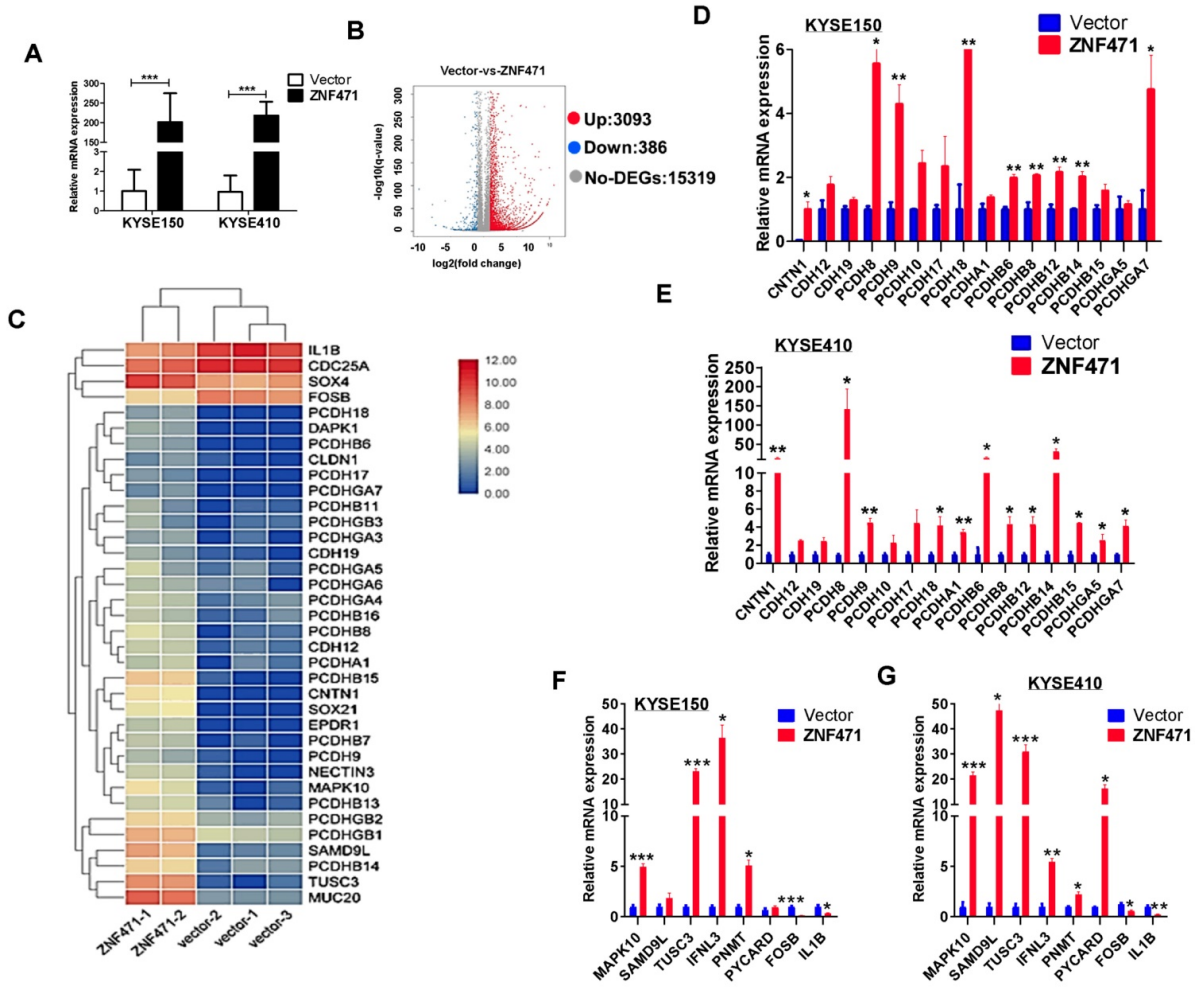
** Bioinformatics analysis of possible mechanism of ZNF471 functions in ESCC cells.** (A) Ectopic expression of ZNF471 in KYSE150 and KYSE410 cells. (B) Entire distribution of differentially expressed genes in vector- and ZNF471-stably transfected KYSE410 cell is shown by a volcano plot. (C) Hierarchical cluster analysis of selected differentially expressed genes in R software is shown as a heat map (gene functional annotation: PCDH family genes, CLDN1 and CNTN1 involved in cell adhesion, MAPK10/JNK3 and PYCARD involved in cell apoptosis, TUSC3 and SAMD9L involved in tumor suppression, and IFNL3 involved in immunity regulation). (D-G) Validation of differentially expressed genes in KYSE150 and KYSE410 cells ectopically expressing ZNF471 by qRT-PCR. Student's test was used. Data are presented as the mean ± SD. *p<0.05, **p<0.01, ***p<0.001.

We performed RNA-sequencing on ZNF471-stably transfected KYSE410 cells (Fig. 6A). The distribution of all differentially expressed genes was shown by a volcano plot, and a total of 3,093 upregulated and 386 downregulated genes were identified (*p*<0.05) (Fig. 6B). The significantly regulated genes identified in both KYSE150 and KYSE410 cells were found to be involved in cell adhesion (PCDH family genes, CLDN1, CNTN1), cell apoptosis (MAPK10/JNK3, PYCARD), tumor suppression (TUSC3, SAMD9L) and immunity regulation (IFNL3). We next performed hierarchical clustering analysis of 36 differentially expressed genes including multiple PCDH family genes, selected on the basis of their expression patterns and classified two major groups (control group vs. ZNF471 group) (Fig. 6C). We further performed qRT-PCR to validate the expression levels of these genes; 24 genes modulated by ZNF471 were verified in both KYSE150 and KYSE410 cells (Fig. 6D-G). These ZNF471-regulated target genes reveal the underlying mechanisms of tumor suppression by ZNF471 in ESCC cells.

### ZNF471-target gene MAPK10/JNK3 suppresses ESCC cell growth and induces their apoptosis

The enriched KEGG pathway plot showed that the greatest number of differentially expressed genes was in the MAPK signaling pathway, among all functional groups (sFig. 4). We used a visual browser, IGV, to further explore the genomic data. We found that MAPK10/JNK3 expression was higher in ZNF471-stably transfected ESCC cells than vector control cells (sFig. 4). Moreover, we found a positive correlation between the expression of ZNF471 and MAPK10 through TCGA database analysis (sFig. 5). These results, together with previous qRT-PCR verification results of differentially expressed genes, indicated that the expression of mitogen activated protein kinase 10 (MAPK10/JNK3) is indeed affected by ZNF471, as verified in both KYSE150 and KYSE410 cells (Fig. 6F, G).

**Figure 7:**
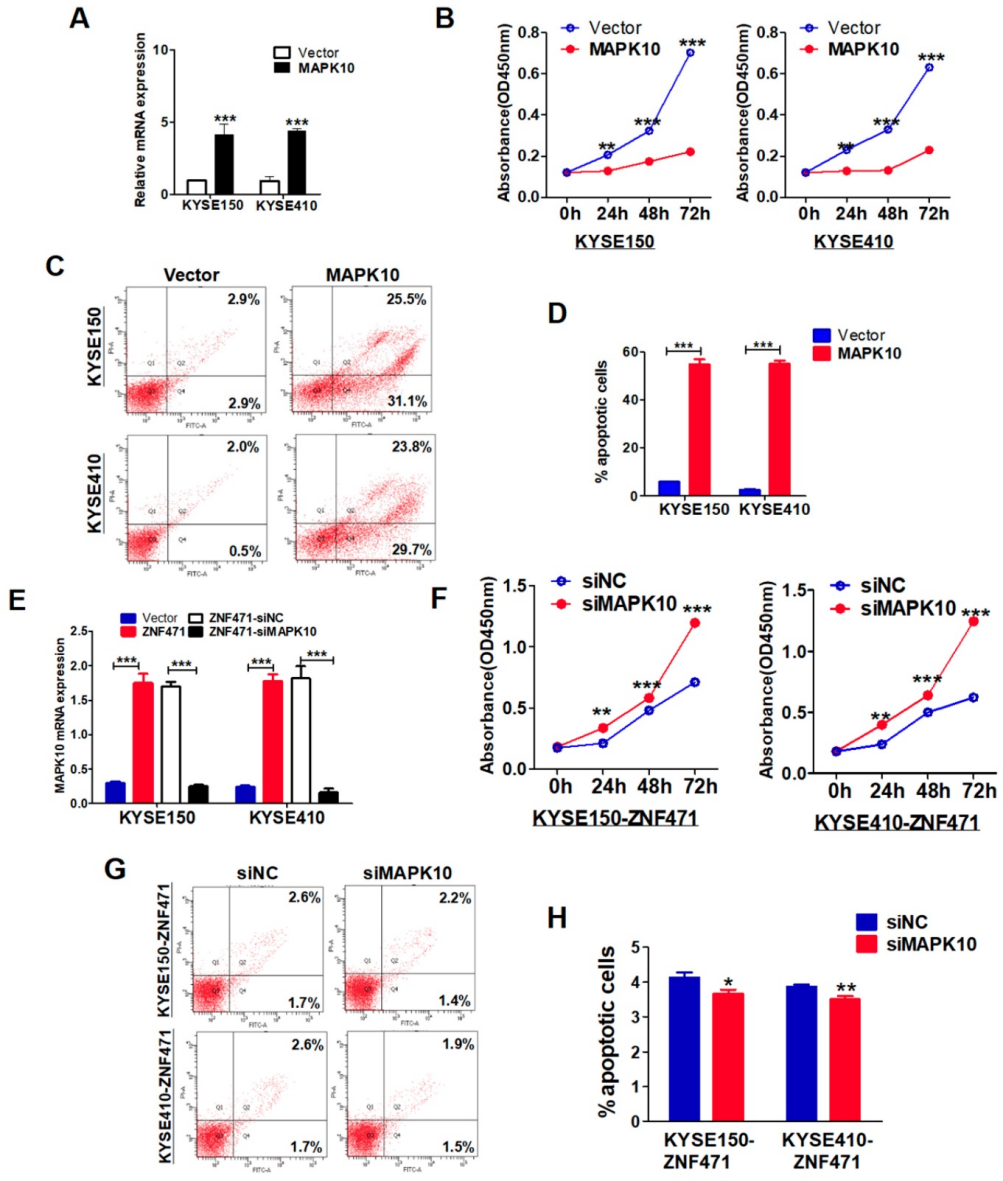
** MAPK10 suppresses ESCC cell proliferation and metastasis.** (A) Ectopic expression of MAPK10 in KYSE150 and KYSE410 cells. (B) Cell viabilities were evaluated at 24, 48 and 72 h after transfection with MAPK10 in KYSE150 and KYSE410 cells. (C, D) Percentages of apoptotic cells in KYSE150 and KYSE410 cells with MAPK10 ectopic expression were evaluated. Cell apoptosis alterations were revealed by histograms. (E) Evaluation of MAPK10-knockdown in ZNF471-infected ESCC cells after transfection with MAPK10 siRNA and siNC by qRT-PCR. (F) Effects of knockdown of MAPK10 on cell viability, as measured by cell viability assays. (G, H) Percentages of apoptotic cells in ZNF471-infected ESCC cells after MAPK10-knockdown were evaluated. Student's test was used. Data are presented as the mean ± SD. *p<0.05, **p<0.01, ***p<0.001.

The specific biological functions of MAPK10/JNK3 in ESCC have not been reported previously. We stably transfected pcDNA3.1-MAPK10 into ESCC cells and found that MAPK10 significantly suppressed cell growth in both KYSE150 and KYSE410 lines, on the basis of cell viability assays (*p*<0.001) (Fig. 7A, B). Moreover, flow cytometry analysis showed that ectopic expression of MAPK10, compared with vector control, significantly increased the numbers of apoptotic cells in KYSE150 and KYSE410 lines (*p*<0.001) (Fig. 7C, D). Furthermore, we conducted MAPK10 rescue experiments in ESCC cells. After knocking down of MAPK10 in ZNF471-stably transfected ESCC cells, the cell proliferation ability was restored, and the numbers of apoptotic cells decreased (Fig. 7E-H). Together, these results demonstrate that the ZNF471-target gene MAPK10/JNK3 does suppress ESCC cell growth.

### Validation of MAPK10/JNK3 as a ZNF471 direct target gene

**Figure 8:**
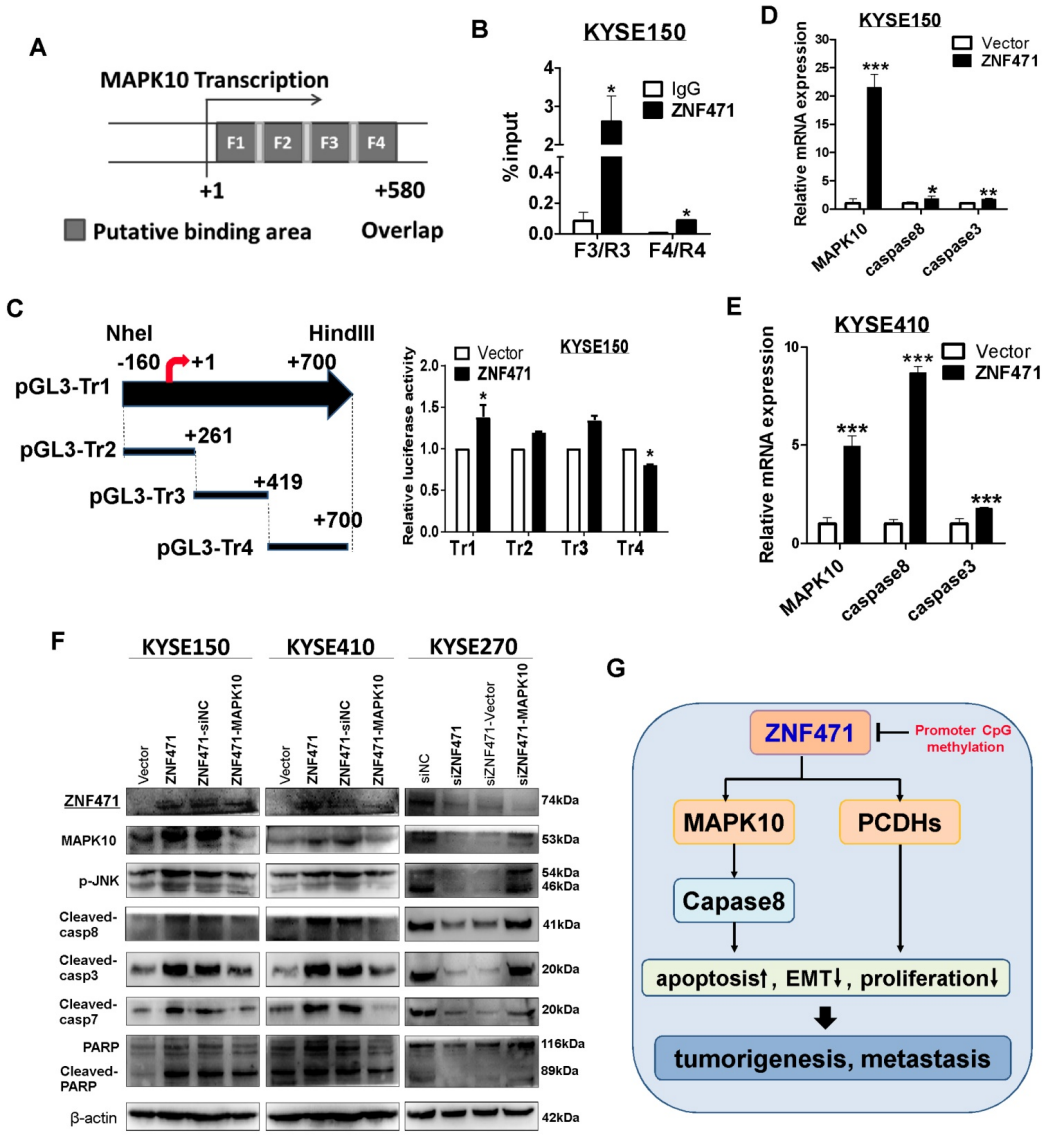
** ZNF471 activates MAPK10/JNK3 signaling and downstream proapoptotic activation in ESCC cells.** (A) Locations of ChIP PCR primers (segment 1(+9-+136), 2(+116-+136), 3(+261-+419) and 4(+400-+580) at the MAPK10 promoter, transcription start site (TSS) is designated as nucleotide +1.F1, Fragments 1;F2, Fragments 2; F3, Fragments 3; F4, Fragments 4.(B) input % of MAPK10 DNA by anti-Flag antibody were determined by ChIP-qPCR. (C) The effect of ZNF471 on MAPK10/JNK3 signaling, as determined by luciferase reporter activity assays. The pLG3-Tr1 plasmid corresponds to all ChIP primer regions, the pLG3-Tr2 truncated plasmid corresponds to the ChIP primer F1/R1 and F2/R2 regions, the pLG3-Tr3 truncated plasmid corresponds to the ChIP primer F3/R3 region, and the pLG3-Tr4 truncated plasmid corresponds to the ChIP primer F4/R4 region. (D, E) qRT-PCR analysis of MAPK10, caspase 8 and caspase 3. (F) Western blot analysis of MAPK10 and downstream effectors. (G) A schematic diagram of the possible mechanism underlying ZNF471 tumor suppression in ESCC cells. Student's test was used. Data are presented as the mean ± SD. *p<0.05, **p<0.01, ***p<0.001.

To further validate MAPK10 as the *bona fide* target gene of ZNF471, we performed chromatin immunoprecipitation (ChIP) quantitative PCR assays on KYSE150 cells, with a Flag antibody and PCR product spanning the identified ZNF471 binding sites. Indeed, ZNF471 was found to directly bind to the *MAPK10* promoter in ESCC cells (data for non-binding sites not shown) (Fig. 8A, B), thus suggesting that MAPK10 is a ZNF471-direct target gene transcriptionally regulated by ZNF471. We also found that ZNF471 may partially regulate MAPK10 through histone H4 acetylation but not histone H2A phosphorylation (sFig. 6). Furthermore, dual-luciferase assays showed that ZNF471 expression significantly activated MAPK10 transcription in both KYSE150 and 293T cells (Fig. 8C, sFig 7). According to the position of these ChIP primers, we designed constructed several truncated plasmids and performed the luciferase assay to confirm the core region of the binding site. We found that the core region of the binding site was segment 4(+419-+700) at the MAPK10 promoter in both KYSE150 and 293T cells (Fig. 8C, sFig 7). We further examined MAPK10 expression after ZNF471 transfection by qRT-PCR and western blotting. The results showed that ZNF471 upregulated the expression of MAPK10 and further activated its downstream effectors including caspase 8, caspase 3, caspase 7, and PARP, at both the transcriptional and protein levels (Fig. 8E-F). These results directly suggested that through direct binding to the MAPK10/JNK3 promoter and promoting its transcription, ZNF471 activated MAPK10 signaling and its downstream effectors, thus further promoting apoptosis and growth inhibition of ESCC cells.

## Discussion

Zinc-finger proteins (ZFPs) are the largest transcription factor family in mammals. Their zinc-finger domains bind to gene promoters and consequently activate or repress the expression of target genes 11. ZFPs can participate in many cell biological processes, including: cell regulation, transcriptional regulation, protein degradation, signal transduction, DNA repair, cell migration, etc. 29. ZFPs are capable of activating or inhibiting the expression of downstream genes by binding to downstream gene promoters 30, 31. The C2H2 type zinc finger structure is the most common type of zinc finger structure among the members. Feng and colleagues describe the role of ZNF339 (OVOL2), a member of the Ovo family of conserved zinc-finger transcription factors, in metastasis and the enrichment of stemness in nasopharyngeal carcinoma. OVOL2 has a strong impact on both metastasis and tumorigenesis in NPC. Low levels of OVOL2 were associated with poor overall survival of NPC patients. Therefore, OVOL2 could serve as a prognostic indicator for cancer patients 32.

KRAB-ZFPs (KRAB-containing zinc finger proteins) are a special class of zinc finger proteins containing a KRAB (Krüppel associated box) domain. Nearly one-third of ZFPs contain a highly conserved Krüppel-associated box (KRAB) motif 33. In previous reports, our group has identified several KRAB-ZFP proteins, including ZNF382, ZNF545 and ZNF331, as important novel TSGs frequently downregulated and methylated in multiple carcinomas 19-23. We also identified another 19q13 KRAB-ZFP, ZNF471, as another member of the KRAB C2H2-type ZFP family methylated in tumors. Frequent ZNF471 methylation has been found in colorectal cancer and tongue squamous cell carcinoma 25, 26. ZNF471 was downregulated and silenced by promoter methylation and ZNF471 suppressed gastric cancer via inhibiting cell proliferation and inducing apoptosis and cell cycle arrest 27. However, to date, its role in ESCC pathogenesis remains unclear. In the present study, we identified ZNF471 as a methylated target silenced in ESCC, and further analyzed its molecular functions in ESCC cells. ZNF471 was found to be highly expressed in normal esophageal tissues but frequently silenced in ESCC. Its loss or downregulation was associated with its promoter methylation, which may serve as a potential biomarker for ESCC. We found that restoration of ZNF471 significantly inhibited ESCC cell growth, through inducing G0/G1 arrest and apoptosis and reversing EMT, and further inhibited ESCC cell invasion and migration. We further found that the underlying mechanisms of tumor suppression by ZNF471 in ESCC cells occur at least partially through its regulated target genes, including the key tumor suppressor MAPK10/JNK3, and multiple other genes involved in cell adhesion (PCDH family genes, CLDN1, CNTN1), tumor suppression (TUSC3 and SAMD9L), apoptosis (PYCARD) and immunity regulation (IFNL3), in both KYSE150 and KYSE410 cells. Collectively, these observations demonstrate that ZNF471 does function as a *bona fide* novel TSG in ESCC.

We identified several ZNF471 downstream effector genes, especially MAPK10/JNK3 and PCDH family genes. MAPK10/JNK3 is a member of the Jun N-terminal kinase subgroup of mitogen-activated protein kinases, which have been implicated in important physiological processes, including apoptosis 34, 35. The activation of stress-induced Jun N-terminal kinase can cause cell death through mitochondrial apoptosis pathways in many cell types. We previously discovered that MAPK10/JNK3 is a novel broad TSG for multiple cancers. In a total of 82 tumor cell lines of different types, MAPK10 expression is frequently downregulated or silenced in non-Hodgkin (94%) and Hodgkin (50%) lymphomas, gastric (60%) and hepatocellular (67%) carcinomas, thus suggesting that MAPK10 has a proapoptotic functions as a TSG 36. According to a previous report, MAPKs play a role in activating caspase cascades, and MAPK10 signaling is linked to the activation of caspase 8 37, 38. Caspase 8 is a major activator of caspase 3, and it amplifies apoptotic signal by directly activating downstream caspases 39. In this study, we found that MAPK10 significantly inhibited ESCC cell growth and induced cell apoptosis. In vitro and in vivo experiments both showed that ZNF471 significantly up-regulated the expression of MAPK10 in ESCC cells. ChIP assays confirmed that ZNF471 directly bound the *MAPK10* promoter, thus suggesting that MAPK10 is a ZNF471 direct target transcriptionally regulated by ZNF471. We also found that ZNF471 may partially regulate MAPK10 through histone H4 acetylation. Unsurprisingly, we found that ZNF471 upregulated the MAPK10 expression and activation of downstream targets such as caspase 8, caspase 3, caspase 7, and PARP at both the transcriptional and protein levels. The direct upregulation of MAPK10/JNK3 expression and activation of downstream proapoptotic targets may be an important mechanism of the tumor suppression of ZNF471 in ESCC cells.

Cadherins are calcium-dependent cell-cell adhesion molecules. Protocadherins (PCDHs) constitute a subfamily of non-classical cadherins 40, 41. The PCDH family consists of three multi-gene clusters (PCDHA, PCDHB and PCDHG) 42. The specific functions of PCDHs in tumorigenesis are still unclear; however, they are assumed to play important roles in the establishment and function of specific cell-cell connections.

Our group previously reported the first evidence that PCDH10 acts as a novel broad TSG that strongly suppresses tumor cell growth, migration, invasion, and colony formation. Interestingly, as a novel broad TSG, and PCDH10 is frequently silenced by promoter methylation in multiple tumors. 43, 44. In addition, other members of the PCDH superfamily have been shown to have tumor suppressor activity, such as PCDH17 and PCDH8 in breast cancer 45, 46, and PCDH20 in nasopharyngeal carcinoma 47. Moreover, previous studies have identified that PCDHs are candidate tumor suppressors that modulate regulatory pathways critical in development and disease, such as canonical Wnt signaling 45, 47, 48. In this study, we found that ZNF471 upregulates multiple PCDH genes, suggesting that ZNF471 may promote adhesion and ligation between ESCC cells and further suppress cell EMT. This possibility is consistent with our observation that ZNF471-stably transfected ESCC cells exhibited adherence and contacts with each other, whereas vector control cells exhibited a scattered pattern. Consistently, we found that ZNF471 inhibited ESCC cell migration and invasion. On the basis of our above observations, we speculate that ZNF471 may inhibit migration and invasion of ESCC cells through upregulating some PCDH genes expression.

In conclusion, we identified and validated ZNF471 as a KRAB-ZFP TSG for ESCC and frequently inactivated by methylation in primary tumors. ZNF471 inhibits ESCC tumorigenesis through inhibiting cell proliferation and promoting apoptosis by activating MAPK10/JNK3 signaling, and suppressing cell invasion and migration through upregulating PCDH expression. The tumor-specific promoter methylation of ZNF471 may be a candidate biomarker for ESCC diagnosis.

## Materials and Methods

### Cell lines, tumor samples and normal tissues

ESCC cell lines used included: EC1, EC18, HKESC1, HKESC2, HKESC3, KYSE30, KYSE70, KYSE140, KYSE150, KYSE180, KYSE220, KYSE270, KYSE410, KYSE450, KYSE510, KYSE520, and SLMT1. Immortalized normal epithelial cell lines (NE1, NE3 and NE083) were also studied^20^, ^49^, ^50^. Some ESCC cell lines and DNA samples from Prof. Gopesh Srivastava; some KYSE cell lines from DSMZ (German Collection of Microorganisms & Cell Cultures); and some normal cell lines from Prof. George Tsao.These cell lines were cultured in RPMI 1640 medium (Gibco-BRL, Karlsruhe, Germany) with 10% fetal bovine serum (Gibco-BRL), at 37°C in a humidified atmosphere containing 5% CO_2_. Primary ESCC tissues, normal esophagus tissues, and adjacent non-cancerous tissues were obtained from patients who had undergone esophagectomy, with no prior treatment before the operation, at the Department of Cardiothoracic Surgery, the First Affiliated Hospital of Chongqing Medical University. All samples were stored at -80°C until evaluation by pathologists. All tumor sample tissues were macro-dissected with 50-70% of tumor cells. All participants provided written consent before enrollment, and the research was approved (ref #2018-083) by Institutional Ethics Committees of the First Affiliated Hospital of Chongqing Medical University. The clinical and pathological features of all participants were obtained, and their statistical characteristics are described in Supplementary Table 1.

### DNA and RNA extraction

Genomic DNA was extracted from cell lines and tissues samples with QIAamp DNA mini kit (Qiagen, Hilden, Germany). Total RNA was extracted with TRIzol reagent (Invitrogen, Carlsbad, CA) 51. The concentrations of DNA and RNA samples were measured through NanoDrop^®^ 2000 spectrophotometry (Thermo Fisher Scientific, Waltham, MA). Sample quality was determined by gel electrophoresis, and samples were stored at -80°C.

### Reverse transcription (RT), semi-quantitative PCR and real-time PCR

RNA was reverse transcribed with a Reverse Transcription System (Promega, Madison, WI) 52. Semi-quantitative RT-PCR was carried out with Go-Taq DNA polymerase (Promega) 52 and performed using a final volume of 10 μL reaction mixture containing 2 μL cDNA, with GAPDH as a control. Amplified PCR products were assayed on 2% agarose gels. Real-time PCR was performed with a SYBR green PCR Master Mix kit (Invitrogen) on ABI 7500 Real-Time PCR system (Applied Biosystems, Foster City, CA )^51^. The relative expression of ZNF471 was estimated with the 2(-Ct) method 53, and all assays were performed in triplicate. GAPDH was used as a loading control. All primers are shown in Supplementary Table 2.

### 5-Aza-2′-deoxycytidine (Aza) and trichostatin A (TSA) treatment

ESCC cell lines were treated with 10 μmol/L 5-aza-2V-deoxycytidine (a demethylation agent) (Sigma-Aldrich, Steinheim, Germany) as previously described 28. For the treatment combining 5-aza-2V-deoxycytidine and trichostatin A (Sigma-Aldrich), cells were treated with 5-aza-2V-deoxycytidine for 3 days and subsequently treated with trichostatin A (an inhibitor of histone deacetylases) (100 ng/mL) for 24 hours.

### Bisulfite treatment, methylation-specific PCR and quantitative methylation specific PCR (qMSP)

To evaluate ZNF471 methylation status, bisulfite modification of DNA, MSP and qMSP were performed as described previously 54-56.Sodium bisulfite-treated DNA was amplified by MSP with methylation-specific primers. MSP analysis revealed no products in any non-bisulfite DNA amplified with MSP primers; thus, it can be considered specific. MSP was performed by using AmpliTaq-Gold DNA Polymerase (Applied Biosystems). Vector was used as a loading control. MSP products were separated on 2% agarose gels (MBI Fermentas, Vilnius, Lithuania) and photographed on a gel imaging system (Bio-RAD Gel Doc XR+, CA). qMSP was performed with the 7500 Real-Time PCR System (Applied Biosystems), and the methylation level of ZNF471 in ESCC cells without Aza and TSA treatments was set as baseline. The methylation-specific primers are shown in Supplementary Table 2.

### Construction of plasmids and stable cell lines

ZNF471 expression vector encoding the full-length open reading frame (ORF) of human ZNF471 was constructed. Briefly, DNA sequences corresponding to ZNF471 ORF were generated by RT-PCR. The sequences of the PCR product were further confirmed by sequencing. The products were then digested with KpnI and BamHI and ligated into pcDNA3.1(+)-Flag-V5 vector, with sequence and orientation confirmed. To construct the pcDNA-MAPK10 plasmid, the MAPK10 full length gene was inserted into a pcDNA3.1(+) framework plasmid. The resultant plasmid was transformed into *E.coli* DH5a cells and sequenced.

DNase was used to process the extracted mRNA before performing RT-PCR. Plasmids were transfected into ESCC cells with Lipofectamine 2000 (Invitrogen, Carlsbad, CA). We screened stable cell by G418 selection. All antibiotic resistant cells were pulled together. Stable cells were verified by both RT-PCR and western blotting.

### Small interfering RNA (siRNA)

ZNF471 and MAPK10 siRNA kits were purchased from OriGene (OriGene Technologies, Rockville, MD). transfections were performed with Lipofectamine 2000 (Invitrogen), with a concentration of 10 nM siRNA. cells were harvested for subsequent assays at 48-72 h after transfection.

### Colony formation assays

Colony formation assays were performed as previously described 52. Stably transfected KYSE150 and KYSE410 cells were plated in six-well plates (800 cells/well) and cultured with various concentrations of G418. The stably transfected ZNF471 cells were obtained using G418 with maintain concentration (200μg/ml for KYSE150, 175μg/ml for KYSE410). After 10-14 d, cells were fixed with 4% PFA and stained with gentian violet (Beyotime Institute of Biotechnology, Shanghai, China). Colonies with >50 cells/colony were photographed with a phase-contrast microscope (Leica DMI4000B, Milton Keynes, Buckinghamshire, UK) and stained and were then counted. Colony counting was done by manual counting with Photoshop software. All experiments were repeated three times.

### Cell viability assays

Stably transfected KYSE150 and KYSE410 cells were seeded in 96-well plates and grown overnight. Cell viabilities were then evaluated with a Cell Counting Kit-8 (Beyotime) at 24, 48, and 72 h 52. All experiments were independently repeated three times.

### Wound healing and Transwell^®^ assays for cell migration

Cell mobility was assessed with scratch wound healing assays. Stably transfected cells (KYSE150 and KYSE410) were cultured in six-well plates until confluent. Cell layers were carefully wounded with pipette tips, and cell migration distance was photographed with a phase-contrast micrographs (Leica DMI4000B) at 0, 12 and 24 h for ESCC cells. Open area of wound healing assay was measured by Image J software.

*In vitro* Transwell^®^ assays were carried out as described previously 57. Transwell^®^ chambers (8-μm pore size, BD Sciences, Bedford, MA) were used with or without Matrigel (BD Biosciences, San Jose, CA) to measure cell migration and invasion, respectively. Cells were collected, washed twice in serum-free medium and added to the upper chamber (5 × 10^4^ cells). The lower chamber contained 700 μl migration-inducing medium with 10% fetal bovine serum (FBS). After incubation for 44 h, cells were fixed with 4% paraformaldehyde for 30 min. and stained for 30 min with crystal violet. Non-migratory cells on the upper side of the filter were wiped off with cotton swabs. Migrated cells were photographed with a phase-contrast microscope (Leica DMI4000B) after fixation and staining and were then counted. Five fields with evenly distributed cells were selected for counting and averaged. All experiments were independently repeated three times.

### Flow cytometry analysis of cell cycle and apoptosis

Cell cycle arrest and apoptosis were assayed by flow cytometry as described previously 52. For cell cycle and apoptosis analysis, stably transfected cells were used. Cells were harvested with trypsin, fixed with ice-cold 70% ethanol and stained with propidium iodide to assay for cell cycle distribution. Apoptosis was assayed with Annexin V-fluorescein isothiocyanate and propidium iodide staining. Data were analyzed with a CELL Quest kit (BD Biosciences). All experiments were independently repeated in triplicate.

### Indirect immunofluorescence determinations

KYSE150 and KYSE410 cells were seeded in 24-well plates on coverslips and allowed to grow overnight; cultures were then infected transiently with pcDNA3.1(+)-Flag-ZNF471-V5. Coverslips were stained through indirect immunofluorescence double staining 57. Briefly, cells were incubated with primary antibodies against E-cadherin (#sc-8426; Santa Cruz Biotechnology, Santa Cruz, CA) or vimentin (#sc-6260; Santa Cruz), or anti-FLAG M2 antibody (#14793; Cell Signaling Technology, Danvers, MA), and then incubated with Alexa Fluor® 594- (Invitrogen Life Sciences, Carlsbad, CA) or FITC-conjugated (Dako, Carpinteria, CA) secondary antibodies against mouse or rabbit IgG. Cells were then counterstained with 4', 6-diamidino-2-phenylindole (DAPI, Roche, Palo Alto, CA, USA) and imaged with a confocal laser scanning microscope (ZEISS LSM 800, Carl Zeiss AG, Oberkochen, Germany). All experiments were independently repeated three times.

### Immunohistochemistry (IHC)

To evaluate the expression levels of ZNF471 in ESCC tissues, tissue microarrays were constructed through paraffin embedding. IHC was performed according to a previously described procedure 58, 59. Anti-Flag was obtained from Abm (#G188; Abm, Richmond, BC), anti-MAPK10 was obtained from Bioss (bs-2997R, Bioss, Beijing, China), and anti-Ki-67 was obtained from Abcam (ab15580; Abcam, Cambridge, UK). Sections were incubated with primary antibody (1:50 dilution) overnight at 4°C and then with secondary antibody (1:2000 dilution) at 37°C for 30 min. Finally, the slides were counterstained with hematoxylin.

### Tumor xenograft model in nude mice

Animal experiments were performed to determine whether ZNF471 inhibits tumor growth *in vivo*. Female nude mice (4-6 weeks of age, weighing 160-190 g) were purchased from the Experimental Animal Center of Chongqing Medical University, China. This study was approved by the Institutional Ethics Committees of the First Affiliated Hospital of Chongqing Medical University (ref #2018-083). Stably ZNF471-expressing KYSE150 or control cells (5×10^6^ cells in 0.1 mL PBS) were injected into the backs of female nude mice (n=10). Tumor diameter was measured every 3 d for 25 d (tumor volume (mm^3^) = length×width^2^×0.5). IHC was used to evaluate the expression of ZNF471, MAPK10 and Ki-67 in nude mouse xenografts. IHC was performed as described previously 58, 59. Anti-Flag was obtained from Abm (#G188; Abm), anti-MAPK10 was obtained from Bioss (bs-2997R, Bioss), and anti-Ki-67 was obtained from Abcam (ab15580; Abcam). Sections were incubated with primary antibody (1:100 dilution) overnight at 4°C, then with secondary antibody (1:2000 dilution) at 37°C for 30 min. Finally, the slides were counterstained with hematoxylin.

### Terminal deoxynucleotidyl transferase (TUNEL) analyses

TUNEL assays were used to detect apoptotic cells in tumor xenograft tissues as described previously. A TUNEL apoptosis detection kit was obtained from Beyotime. Images were captured with a Leica LSM 400 laser scanning microscope (Leica, Wetzlar, Germany), and the rate of apoptosis was quantified with Image Pro Plus software (Media Cybernetics, Rockville, MD).

### Western blotting

Western blot assays were performed as described previously 51. Stably transfected KYSE150 and KYSE410 cells were washed three times with ice-cold PBS and then lysed in lysis buffer (Beyotime) containing a protease inhibitor cocktail (Sigma-Aldrich). Protein samples of 40 μg were separated by 10-12% sodium dodecyl sulfate polyacrylamide gel electrophoresis and then transferred to polyvinylidene difluoride membranes (Merck Millipore, Billerica, MA). After being blocked with 5% nonfat milk, the membranes were incubated at 4°C overnight with the primary antibodies to the following: ZNF471 (HPA066695; Sigma-Aldrich), ZNF471 (Y158334; Abm), p21 (sc-126; Santa Cruz), p27 (sc-393380; Santa Cruz), cleaved caspase 3 (#9661; Cell Signaling Technology), cleaved caspase 8 (#8592; Cell Signaling Technology), cleaved PARP (#5625; Cell Signaling Technology), Cyclin D1 (#1677; Epitomics, Burlingame, CA,), E-cadherin (#1702-1; Epitomics), vimentin (#2707-1, Epitomics), N-cadherin (ab98952; Abcam), SNAIL (#3897; Cell Signaling Technology), anti-Flag (#G188; Abm), anti-Flag M2 (#14793; Cell Signaling Technology), MAPK10 (ab126591; Abcam), p-JNK (WL01295, Wanleibio, China), and β-actin (sc-47778; Santa Cruz; loading control). Dilution of primary and secondary antibodies was performed according to the manufacturers' recommendations. Membranes were visualized with Eletrochemiluminescence Plus Detection Reagents (RPN2132; GE Healthcare Life Science, Buckinghamshire, UK). All assays were independently repeated three times.

### Dual-luciferase reporter assays

Reporter gene plasmids were generated with the pGL3/Basic plasmid as a framework, which expresses the firefly luciferase reporter. Promoter regions of genes were amplified by PCR and cloned into the pGL3/Basic plasmid with a seamless cloning kit (D7010S, Beyotime, Beijing, China), and further confirmed by sequencing.

For ZNF471 reporter assays, 293T or KYSE1500 cells were co-transfected with ZNF471, pGL3-gene and pRT-LK (80:1 ratio). The promoter-less pGL3/Basic vector was used as a negative control. Renilla luciferase was used as an internal control to assess transfection efficiency. At 24 h post-transfection, cells were lysed in 200 μl lysis buffer. For the luciferase read-out, 20 μl of sample was detected with a Dual luciferase reporter assay kit (Promega), and additional 100 μl substrate was added. Light emission was quantified with a standard manual luminometer (Infinite M200 PRO, Tecan, Austria). Five independent transfections were performed in each case.

### Chromatin Immunoprecipitation (ChIP) assays

Chromatin immunoprecipitation (ChIP) was performed according to the manual of the SimpleChIP® Enzymatic Chromatin IP Kit (#9003, Cell Signaling Technology). Stably ZNF471-expressing KYSE150 cells and vector-expressing cells were washed with ice-cold PBS devoid of Ca_2_^+^ and Mg_2_^+^ and supplemented with a protease inhibitor cocktail (PIC, P8340, Sigma-Aldrich). DNA and protein were cross-linked with 1% formaldehyde at 37°C for 10 min at room temperature. Reactions were terminated with 0.125 M glycine for 5 min. Cells were then lysed in lysis buffer (20 mM Tris-HCl, pH 8.0, 50 mM NaCl, 5 mM CaCl_2_, 1% Triton X-100, PIC) for 10 min, and micrococcal nuclease was added for 20 min at 37°C. Cells were then sonicated on ice for six sets of 10 sec pulses at 40% amplitude with an ultrasonic cell disruptor (JY88-IIN, Scientz, China) at an interval of 1 min, to generate 200-900 bp DNA fragments. A 10 μl sample of the diluted chromatin lysates was removed to serve as an input sample. diluted samples were incubated with rabbit anti-Flag M2 (5 μl) (#14793, Cell Signaling Technology), anti-histone H4 acetylation (5 μl) (NM_175054, Merck Millipore, Billerica, MA, USA) or anti-histone H2A phosphorylation (5 μl) (05-636, Upstate Biotechnology, Lake Placid, NY, USA) overnight at 4°C, and this was followed by capture with protein A/G magnetic beads (#9006, Cell Signaling Technology) for 2 h. Rabbit anti-histone H3 antibody (#4620, Cell Signaling Technology) and normal rabbit IgG (#2729, Cell Signaling Technology) were used as positive and negative controls, respectively. The complexes were precipitated, washed, and eluted as recommended. After DNA-protein cross-linkages were incubated with 6 μl of 5 M NaCl and 2 μl of proteinase K at 65°C for 2 h, DNA was purified with spin columns for further use in qRT-PCR (RT-PCR) analyses. An equivalent volume of each sample was used as a template for PCR amplification. Specific primers are listed in Supplementary Table 2. The experiment was repeated three times independently.

### RNA sequencing (RNA-Seq)

RNA-Seq was performed by BGI TechSolutions Co., Ltd (Shenzhen, China). The RNA integrity number was assessed with an RNA Nano 6000 Assay Kit for the Agilent Bioanalyzer 2100 system (Agilent Technologies, Santa Clara, CA). A total amount of 3 μg RNA per sample was used as input material for the construction of cDNA libraries, whose quality was assessed on the Agilent Bioanalyzer 2100 system. The library preparations were sequenced on the BGISEQ-500 RNA-seq platform (BGI), and 50-bp single-end reads were generated. Genes with an adjusted *p*<0.05 and identified with DESeq 60 were considered to be differentially expressed.

### Pathway enrichment analysis

The functional enrichment of differentially expressed genes was analyzed with GO classification 61 and Kyoto Encyclopedia of Genes and Genomes (KEGG) pathways 62 from the Database for Annotation, Visualization and Integrated Discovery (DAVID, http://david.abcc.ncifcrf.gov/) 63. The level of significance was *p*<0.05.

### Statistical analysis

All data are representative of three independent experiments and are presented as the mean ± SD. SPSS20.0 software (Chicago, IL) was used for statistical analyses. Student's t-test and Dunnett's t-test were used to analyze the significance of differences between the experimental and control values. χ^2^ and Fisher exact tests were used to compare the ZNF471 promoter methylation status with clinicopathological features. We used technical replicates for statistical analyses. We performed p-value correction by FDR (false discovery rate) in the RNA-seq data analysis. For all tests, *p*<0.05 was considered statistically significant.

## Supplementary Material

Supplementary figures and tables.

## Figures and Tables

**Table 1:** The promoter methylation of ZNF471 in primary ESCC

Samples	ZNF471 methylation status		% of methylation
	Methylated	Unmethylated	
ESCC (N=147)	92	55	62.6% (92/147)
Adjacent non-cancerous tissues (N=89)	22	67	24.7% (22/89)
EN (N=3)	0	3	0% (0/3)

ESCC Esophageal squamous cell carcinoma, EN Normal esophagus tissues
