# Supplementary material for: 19q13 KRAB zinc-finger protein ZNF471 activates MAPK10/JNK3 signaling but is frequently silenced by promoter CpG methylation in esophageal cancer
Source: Theranostics. 2020 Jan 12;10(5):2243–59. doi: 10.7150/thno.35861 (PMC7019175; doi:10.7150/thno.35861)
Supplement: Supplementary file 1 — Supplementary figures and tables. [file thnov10p2243s1.pdf]

Suppl. Table 1. Association between *ZNF471* methylation and clinicopathological parameters of ESCC patients.

**Clinicopathological features**	**Numbers (N=79)**	**ZNF471 methylation status**		***p* value**
		Methylated	Unmethylated	
**Age**				0.779
＜63	46	25	21	
≥63	33	19	14	
Gender				0.613
Male	63	36	27	
Female	16	8	8	
**Tumor Location**				0.970
Upper	16	11	5	
Middle	46	20	26	
Lower	16	12	4	
Unknown	1	1	0	
**Differentiation**				0.649
Moderately/Well	72	40	32	
Poorly	7	4	3	
**Tumor Grade**				0.457
T1–2	28	14	14	
T3–4	51	30	21	
**Tumor Size**				0.571
>5.0 cm	6	2	4	
≥2.0 cm ≤5.0 cm	64	37	27	
<2.0 cm	4	2	2	
Unknown	5	3	2	
**Tumor Stage**				0.971
I/II	54	30	24	
III/IV	25	14	11	
**Lymph Nodes Metastasis**				0.710
Positive	22	13	9	
Negative	57	31	26	
**Distant Metastasis**				0.376
Positive	1	1	0	
Negative	78	43	35	

χ^2 and Fisher exact tests were used.

Suppl. Table 2. Primers used in this study

PCR	Primer	Sequence (5'-3')	Product size (bp)	PCR cycles	Annealing temp. (℃)
RT-PCR	ZNF471F	GAGATGACGAGTGAGATGAC	194bp	32	55
	ZNF471R	TGACTTCCCATCTGCTTCTC			
	GAPDHF	CCAGCAAGAGCACAAGAGGAA	114bp	23	55
	GAPDHR	GGTCTACATGGCAACTCAAGG			
qRT-PCR	MAPK10F	CATCCCTACATCAACGTCTG	249bp		60
	MAPK10R	GGAGGAGATGTCATTGACAG			
	SAMD9LF	GCTAGAAGCTCTGAGAGCAGA	116bp		60
	SAMD9LR	TGCTGCAGTAGGAAGGCATA			
	TUSC3F	GTATGGTGGACTATGATGAGGG	120bp		60
	TUSC3R	TGGAGGTCAAAAGTATCAGCTC			
	IFNL3F	GCTGCCACATAGCCCAGTTC	157bp		60
	IFNL3R	CTCACCTGCAGCTGCCTCA			
	PNMTF	CTCATCGACATTGGTTCAGGC	175bp		60
	PNMTR	TGAGGCAGGCATGTTGGCTG			
	PYCARDF	CAAGCTGAAGCTGCTGTCG	162bp		60
	PYCARDR	CCATGTCGCGCAGCACGT			
	FOSBF	GGCGACCCCTTCCCCGTTGT	102bp		60
	FOSBR	ACTGCCGCTGGTGCGTTGG			
	IL1BF	GCCTGAAGCCCTTGCTGTAGT	51bp		60
	IL1BR	GCGGCATCCAGCTACGAAT			
	CNTN1F	GTTGCTGGTCAGTCATCTTGTG	140bp		60
	CNTN1R	TGGTATTGATTGGCTGCTCTTC			
	CDH12F	AGCAGACTTTAGCCACAGAGC	182bp		60
	CDH12R	AGTGCCCTCTCCCTTGTCTA			
	CDH19F	GTCAAGCAGCCAGTGCGATCT	169bp		60
	CDH19R	CAGCTCCAGCTCCCAAAAGC			
	PCDH8F	CGACAGCGATTCCGACATCA	273bp		60
	PCDH8R	CTCTGCAGCCCCACTGTCTT			
	PCDH9F	CTGTTGGCTGCTCTGATTGC	148bp		60
	PCDH9R	CTGTGGCAGCATTGATGTGAG			
	PCDH10F	ACTGCTATCAGGTATGCCTG	219bp		60
	PCDH10R	GTCTGTCAACTAGATAGCTG			
	PCDH17F	TGGAGGAGAGGAACGCCATG	299bp		60
	PCDH17R	AACAAACTGCTGCCTGCTGC			
	PCDH18F	CACGATGTACTGGGCAAGAAT	138bp		60
	PCDH18R	GGCTCGAAATCGAACAGTAG			

	PCDHA1F	CTCCAAGTCTTAACACGTCAGA	124bp	60
	PCDHA1R	CTGTGCATGCCTGCTCTCAG		
	PCDHB15F	GACCGTCTGTTAATTTTGTCTTCC	129bp	60
	PCDHB15R	CAGACAAAGGAAATGAGTAACAT		
	PCDHB6F	AGCCATTCATACTTATGCATATTT	124bp	60
	PCDHB6R	GCTCACAAACTAATGTGTTGACA		
	PCDHB8F	GGAGCCTGTCTCAGAACTATCAGT	124bp	60
	PCDHB8R	ACTGGTTTCAGGAACTGGAACTCA		
	PCDHB12F	GGGAGAACCTAGAAGCCATTCAAC	124bp	60
	PCDHB12R	CGCAGTTGTGGGAAACGT		
	PCDHB14F	GGACATTTTGGAGAGAACTG	124bp	60
	PCDHB14R	GTAAGGCCTTCTAAAAGATAATTG		
	PCDHGA5F	GCGAGCCTCTTCTGATGTCTGATA	124bp	60
	PCDHGA5R	GAGAAACGCCAGTCCGTGTTG		
	PCDHGA7F	TGCTAACATCCGTAGATTTTCAGG	124bp	60
	PCDHGA7R	GAGAAACGCCAGTCCGTGTTG		
**MSP**	ZNF471m44	TTTTGTTTTCGTTTTTTTCGTTC	223bp	60
	ZNF471m6	ACGCGACTAAACCTTCGCG		
	ZNF471u44	GTTTTGTTTTTGTTTTTTTTGTTT	228bp	58
	ZNF471u6	AAAAACACAACTAAACCTTCACA		
**ChIP assay**	MAPK10proF1	GAGAAGAGGAGCAGCATCTC	127bp	
	MAPK10proR1	AGGCGTCGAGCACAGTAGGG		
	MAPK10proF2	CCCTACTGTGCTCGACGCCT	164bp	
	MAPK10proR2	GTGACACACCATGCTCACG		
	MAPK10proF3	CGTGAGCATGGTGTGTCAC	158bp	
	MAPK10proR3	AGCTCGGGGTGGGAGATCT		
	MAPK10proF4	AGATCTCCCACCCCGAGCT	180bp	
	MAPK10proR4	CGCTCGGTCACCAACCTGA		
**Dual-luciferase report assay**	MAPK10procF	CTAGCCCGGGCTCGAGCGAAAGC AGAGAGAGGAGGAA	860bp	
	MAPK10procR	CCGGAATGCCAAGCTTCAGGCGCT AGGAACTCTGC		

Supplementary Figure legends

Supplementary Fig 1. Correlation analysis of expression and promoter methylation status of ZNF471 in ESCC tissue samples from The Cancer Genome Atlas (TCGA).

Supplementary Fig 2. Cell migration abilities of ESCC cells, evaluated by wound healing assays. Photographs were captured at 0, 12 and 24 h. Representative wound healing ratio is shown. Scale bars: 200 μm.

Supplementary Fig 3. Expression and location of E-cadherin and Vimentin markers in ESCC cells, as detected by confocal microscopy. Scale bars: 10 μm.

Supplementary Fig 4. (A, B) MAPK10 expression level in ZNF471-stably transfected KYSE410 cells, shown by visualization graphs and histogram (C) KEGG pathway categories of differentially expressed genes. The rich factor represents the proportion of differentially expressed genes within a specific term, and the size of the point represents the number of related differentially expressed genes. The q-value is the calibrated p value.

Supplementary Fig 5. Correlation analysis of expression of ZNF471 and MAPK10 in ESCC tissue samples from The Cancer Genome Atlas (TCGA).

Supplementary Fig 6. % input of MAPK10 DNA by anti-histone H4 acetylcholine antibody and anti-histone H2A phosphorylation were determined by ChIP-qPCR.

Supplementary Fig 7. The effect of ZNF471 on MAPK10/JNK3 signaling, as determined by luciferase reporter activity assays in 293T cells. The pLG3-Tr1 plasmid corresponds to all ChIP primer regions, the pLG3-Tr2 truncated plasmid corresponds to the ChIP primer F1/R1 and F2/R2 regions, the pLG3-Tr3 truncated plasmid corresponds to the ChIP primer F3/R3 region, and the pLG3-Tr4 truncated plasmid corresponds to the ChIP primer F4/R4

region.

sFig 1.

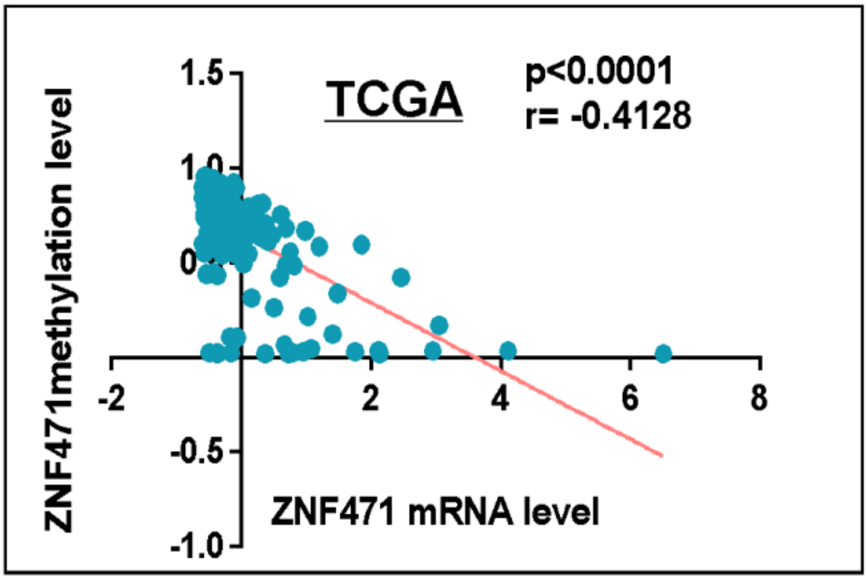

sFig 2.

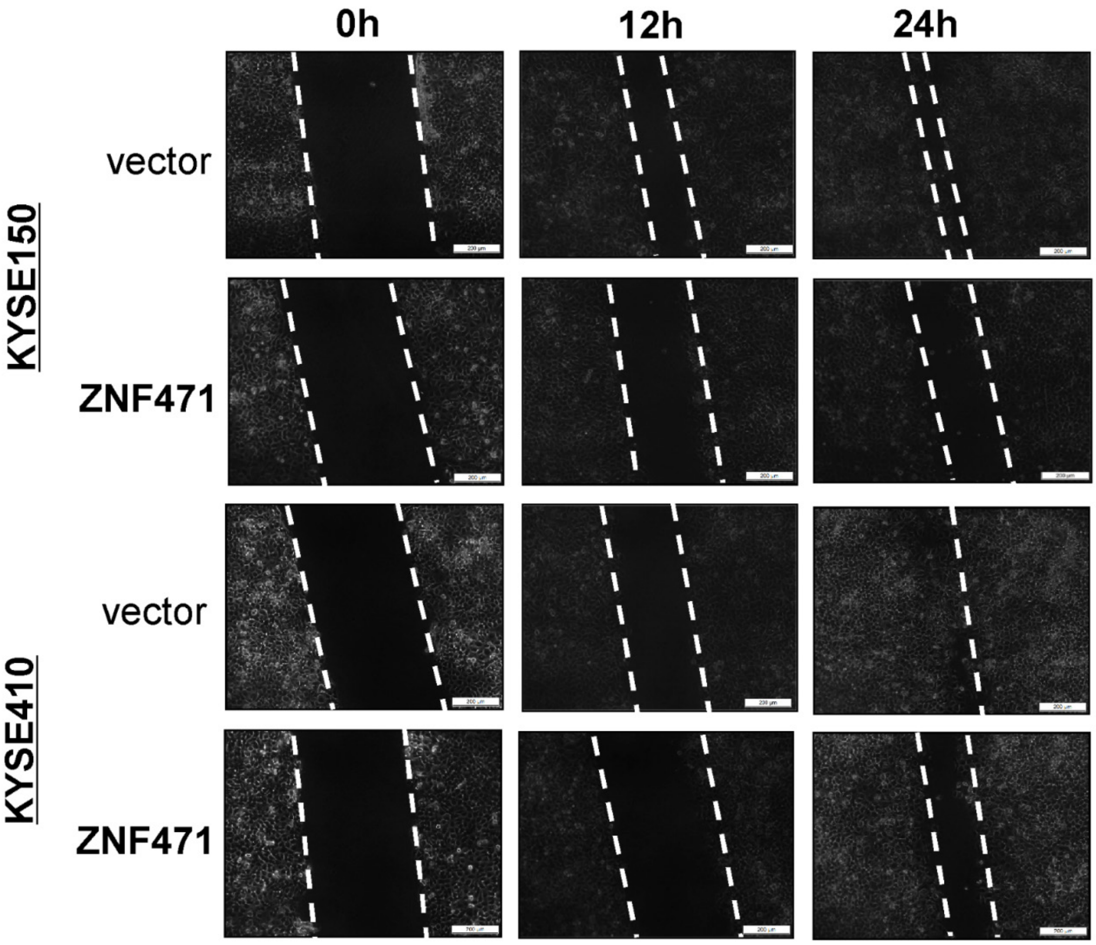

sFig 3.

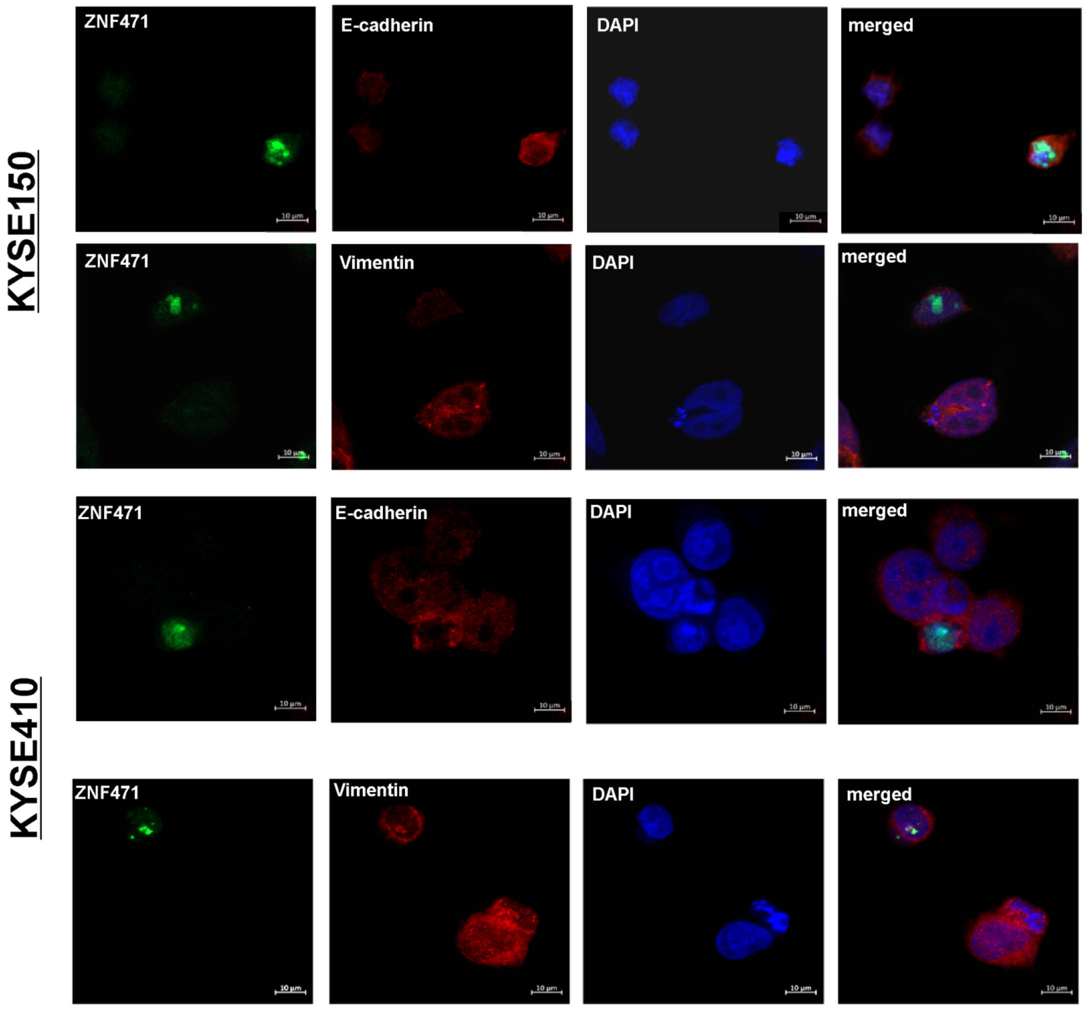
ZNF471
E-cadherin
DAPI
merged
ZNF471
Vimentin
DAPI
merged
ZNF471
E-cadherin
DAPI
merged
ZNF471
Vimentin
DAPI
merged
KYSE150
KYSE410

sFig 4.

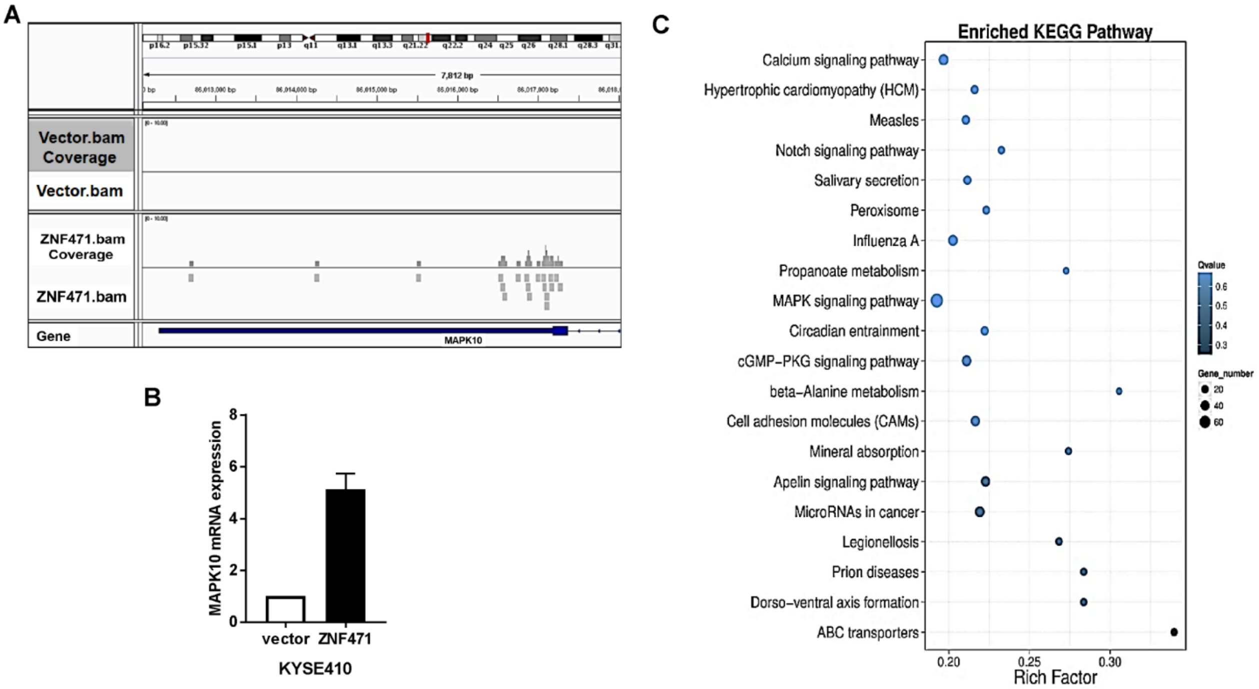
A
Vector.bam Coverage
Vector.bam
ZNF471.bam Coverage
ZNF471.bam
Gene
MAPK10
7,812 bp
B
MAPK10 mRNA expression
8
6
4
2
0
vector
ZNF471
KYSE410
C
Enriched KEGG Pathway
Calcium signaling pathway
Hypertrophic cardiomyopathy (HCM)
Measles
Notch signaling pathway
Salivary secretion
Peroxisome
Influenza A
Propanoate metabolism
MAPK signaling pathway
Circadian entrainment
cGMP-PKG signaling pathway
beta-Alanine metabolism
Cell adhesion molecules (CAMs)
Mineral absorption
Apelin signaling pathway
MicroRNAs in cancer
Legionellosis
Prion diseases
Dorso-ventral axis formation
ABC transporters
0.20
0.25
0.30
Rich Factor
Qvalue
0.6
0.5
0.4
0.3
Gene_number
20
40
60

sFig 5.

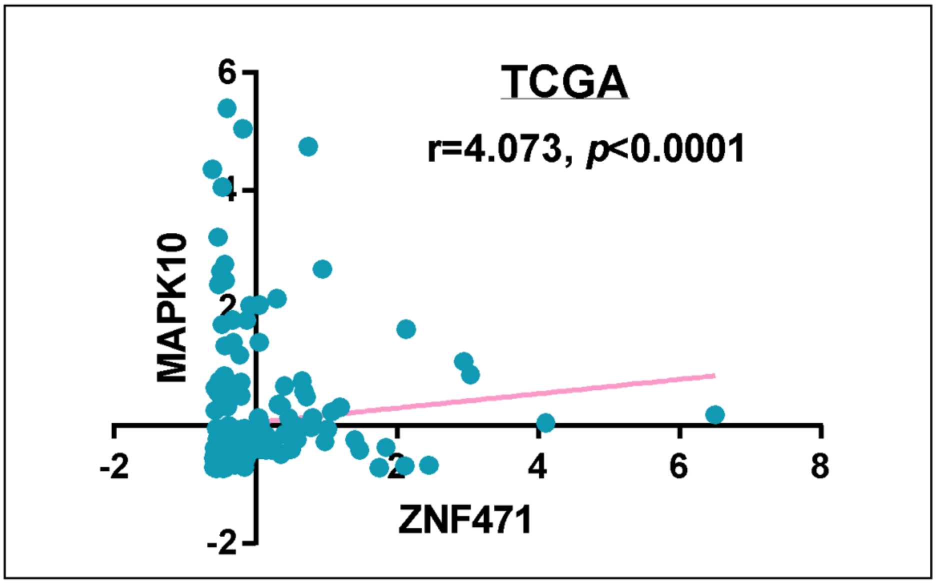

sFig 6.

MAPK10 proF2/R2

MAPK10 proF3/R3

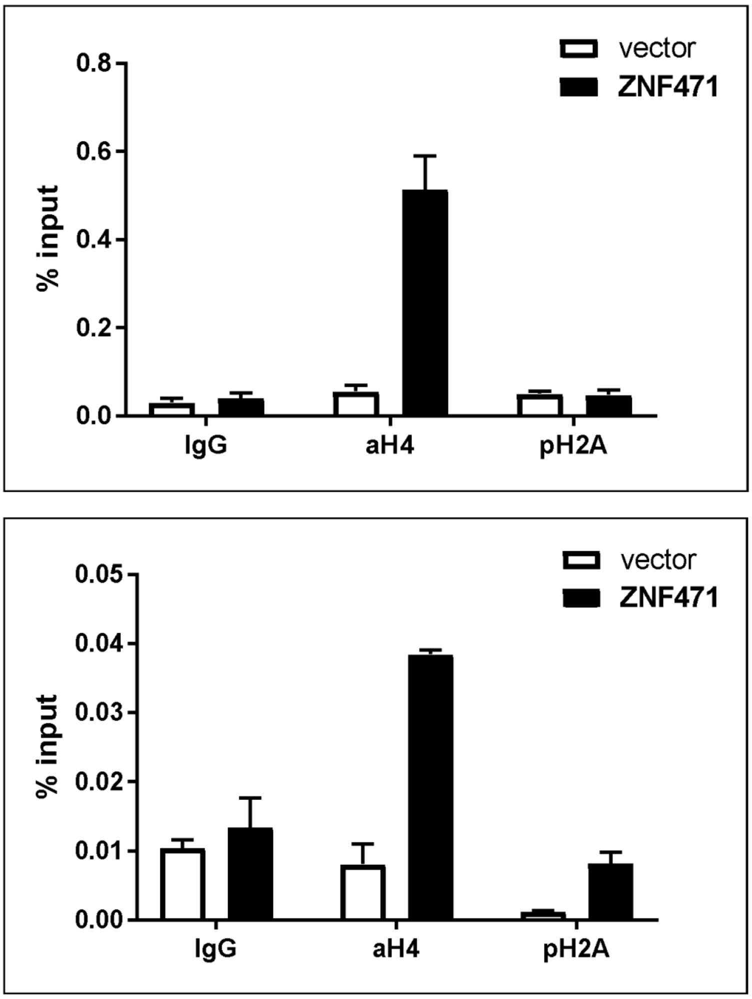

sFig 7.

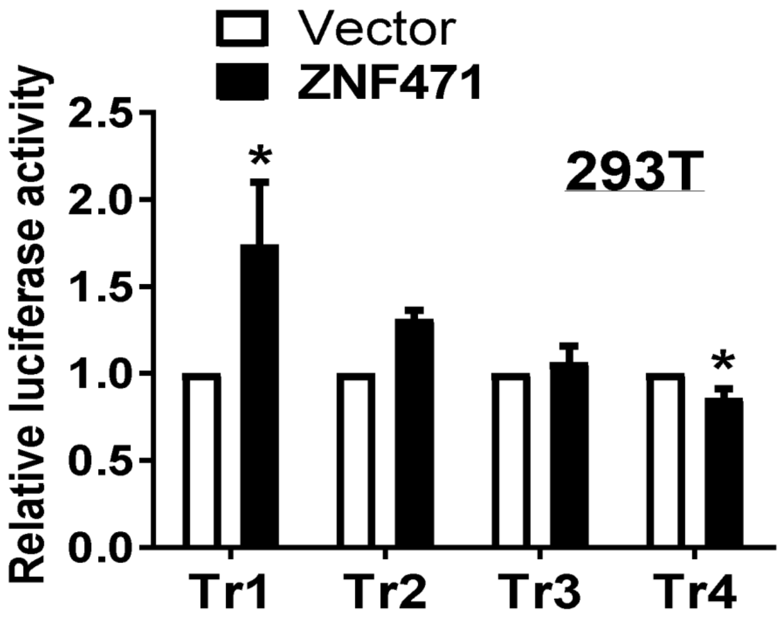
Vector
ZNF471
Relative luciferase activity
2.5
2.0
1.5
1.0
0.5
0.0
*
*
293T
Tr1
Tr2
Tr3
Tr4
